# Role of the YAP Oncoprotein in Priming Ras-Driven Rhabdomyosarcoma

**DOI:** 10.1371/journal.pone.0140781

**Published:** 2015-10-23

**Authors:** Katherine K. Slemmons, Lisa E. S. Crose, Erin Rudzinski, Rex C. Bentley, Corinne M. Linardic

**Affiliations:** 1 Department of Pharmacology and Cancer Biology, Duke University Medical Center, Durham, North Carolina, United States of America; 2 Department of Pediatrics, Duke University Medical Center, Durham, North Carolina, United States of America; 3 Department of Laboratories, Seattle Children’s Hospital, Seattle, Washington, United States of America; 4 Department of Pathology, Duke University Medical Center, Durham, North Carolina, United States of America; Institute of Molecular and Cell Biology, Biopolis, UNITED STATES

## Abstract

Rhabdomyosarcoma (RMS), a cancer characterized by features of skeletal muscle histogenesis, is the most common soft tissue sarcoma of childhood and adolescence. Survival for high-risk groups is less than 30% at 5 years. RMS also occurs during adulthood, with a lower incidence but higher mortality. Recently, mutational profiling has revealed a correlation between activating Ras mutations in the embryonal (eRMS) and pleomorphic (pRMS) histologic variants of RMS, and a poorer outcome for those patients. Independently, the YAP transcriptional coactivator, an oncoprotein kept in check by the Hippo tumor suppressor pathway, is upregulated in eRMS. Here we show that YAP promotes cell proliferation and antagonizes apoptosis and myogenic differentiation of human RMS cells bearing oncogenic Ras mutations in cell culture studies *in vitro* and in murine xenografts *in vivo*. Pharmacologic inhibition of YAP by the benzoporphyrin derivative verteporfin decreased cell proliferation and tumor growth *in vivo*. To interrogate the temporal contribution of YAP in eRMS tumorigenesis, we used a primary human cell-based genetic model of Ras-driven RMS. Constitutively active YAP functioned as an early genetic lesion, permitting bypass of senescence and priming myoblasts to tolerate subsequent expression of hTERT and oncogenic Ras, which were necessary and sufficient to generate murine xenograft tumors mimicking RMS *in vivo*. This work provides evidence for cooperation between YAP and oncogenic Ras in RMS tumorigenesis, laying the foundation for preclinical co-targeting of these pathways.

## Introduction

Rhabdomyosarcoma (RMS) is the most common soft tissue sarcoma of childhood and adolescence, accounting for 7% of soft tissue malignancies in these age groups [1]. The embryonal histologic subtype (eRMS) accounts for the majority of cases, with excellent survival for low and intermediate-risk groups [1]. Pleomorphic RMS (pRMS) is more common in adults and is associated with a very poor outcome [2]. Despite intergroup clinical trials, survival for high-risk RMS has remained low at 30% and has not improved in 30 years [3]. Understanding the molecular lesions in these high risk groups will be critical for improving outcomes.

Beginning in 1989, mutations of the *RAS* oncogene (*K*, *N*, *and H* isoforms) were sporadically noted in eRMS [4–6]. We and others used model systems to investigate and determine the contributions of oncogenic Ras to eRMS tumorigenesis. Since primary cell-based models permit the study of gene combinations underlying tumorigenesis [7], we constructed a genetically-defined model of eRMS and found that serial stable expression of three oncogenic cDNAs (the DNA tumor virus *SV40* early region encoding large T and small t antigens, the catalytic subunit of telomerase *h* *TERT*, and oncogenic *H* *R* *ASG12V*, *“THR”*) was necessary and sufficient to convert normal primary human skeletal muscle myoblasts (HSMMs) to tumorigenic cells mimicking eRMS [8]. Complementary studies in lower vertebrates including zebrafish and mice have further illuminated the role of oncogenic Ras in eRMS [9, 10] and pRMS [9, 11, 12]. During the last several years, deep-sequencing technologies have permitted a more precise profile of Ras mutations in RMS. These mutations are relatively common in RMS, occurring in about 30% of cases, and are associated with high risk patients [13, 14]. Thus this subset of RMS tumors can be termed “Ras-driven RMS.” However, since stable expression of oncogenic Ras in primary human myoblasts activates senescence checkpoints [8], we reasoned that other genetic changes must occur prior to and permit tolerance of oncogenic Ras.

The Hippo pathway is an evolutionarily conserved tumor suppressor network that, among other functions, controls and limits organ size during both embryogenesis and tissue regeneration [15]. Genomic amplifications and mutations of Hippo pathway components (or more often of proteins that modulate Hippo pathway signaling) are emerging as important genetic lesions in human tumorigenesis, although there are only a few reported cases in RMS [16], including copy number gains of YAP or TAZ [17, 18]. However, recently we and others have found the YAP oncoprotein, a transcriptional co-activator situated at the terminus of the Hippo pathway and ordinarily kept in check by this signaling cascade, to be upregulated in RMS [17, 19]. YAP binds the TEAD family of transcription factors to regulate pro-growth and anti-apoptotic genes. In genetically engineered mouse models (GEMMs), conditional expression of an activated form of YAP (S127A) in satellite cells generated eRMS with high penetrance, after injury [17]. This suggests that YAP is not only upregulated in eRMS, but critical for its initiation. However, since in human tumors multiple oncogenic events are required for tumorigenesis [20], and since eRMS tumors often have Ras mutations, we hypothesized that YAP could cooperate with oncogenic Ras to initiate eRMS tumorigenesis.

Here we investigate the role of YAP in Ras-driven RMS cell lines, showing that YAP is critical for tumor maintenance, since it supports RMS cell proliferation, survival, and inhibition of differentiation. We use a human primary cell-based approach to understand the temporal role of YAP in a Ras-driven model of RMS. By substitution into our established genetically defined model, we find that YAP enables bypass of the senescence checkpoint, then provides tolerance to expression of oncogenic Ras. This model is a novel tool to explore the role of YAP in a human primary cell system. Additionally, these studies lay the groundwork for future investigations to understand the interaction between YAP and Ras signaling in eRMS and pRMS, a critical step in designing rational therapies.

## Materials and Methods

### Generation of Cell Lines and Constructs

Human RMS cell lines RD [21] and SMS-CTR [22] were gifts from Tim Triche (Children’s Hospital of Los Angeles, CA, USA) in 2005 and Brett Hall (Columbus Children’s Hospital, OH, USA) in 2006, respectively, and cultured as described [23]. RD and SMS-CTR cells express oncogenic mutations of *NRAS* and *HRAS*, respectively [24–26]. Cell line authentication was performed in July 2014 using STR analysis (Promega PowerPlex 18D) conducted by the DNA Analysis Facility at Duke University (Durham, NC, USA). Human skeletal muscle myoblasts (HSMM, Lonza) were cultured as described [19] and YAP shRNA constructs were previously described [27, 28]. pQCXIH-Flag-YAP-S127A was a gift from Kunliang Guan (Addgene plasmid # 33092) [29] and pBABE YAP1 WT was a gift from Joan Brugge (Addgene plasmid # 15682) [30].

### Quantitative Real Time PCR and Semi-quantitative PCR

PCR was performed as described [19]. Primer sets for this work can be found in S1 Table.

### Immunoblotting

Immunoblotting was performed as described, using a range of 10 to 100μg of lysate per sample [31]. The following antibodies were used for immunoblotting: anti-YAP (Cell Signaling #4912, 1:1000), anti-pan-Ras (Calbiochem #OP40, 1:1000), anti-cleaved caspase 3 (Cell Signaling #9661, 1:1000), anti-TAZ (Cell Signaling #4883, 1:1000) and anti-actin (Sigma #A2066, 1:1000 or Sigma #A5441, 1:5000).

### Growth Curves and BrdU

Cell growth was analyzed by manual cell counting on a hemocytometer following Trypan blue staining, performed in triplicate. BrdU assays to measure cell proliferation were performed in five replicates as described [19].

### Differentiation Assays

Differentiation assays and MF20 staining were performed as described [32]. The MF20 antibody recognizes all isoforms of myosin heavy chain in differentiated skeletal muscle and was deposited to the Developmental Studies Hybridoma Bank by Fischman, D.A. (DSHB Hybridoma Product MF 20). Positively and negatively stained cells were counted manually with the aid of cell counting software (ImageJ, NIH). Five images were counted per condition.

### Senescence Assays

Performed as described except in 24-well plates and in triplicate [19].

### Ethics Statement

All animal studies were conducted in accordance with policies set forth by the Duke University Institutional Animal Care and Use Committee (IACUC). Our protocol was approved by the Duke IACUC (Protocol Registry Number A183-14-07). Euthanasia was performed by CO2 and bilateral thoracotomy.

### Mouse Xenograft Studies

For the YAP suppression study, 1x10^6^ SMS-CTR cells stably expressing non-targeting, YAP_sh3, or YAP_sh4 constructs were resuspended in Matrigel (BD Biosciences) and implanted subcutaneously into the flanks of immunodeficient SCID/*beige* mice as described [19, 31]. For the YHR model study, 10x10^6^ YHR, YHV, or SMS-CTR cells were injected similarly. For the verteporfin study, 2x10^6^ SMS-CTR cells were injected as described. Mice were monitored twice weekly, and tumors were measured using calipers. Tumor volume was calculated as [((width*length)/2)3]/2. Mice were sacrificed upon reaching an IACUC-defined maximum tumor burden or decline in health. Tumors were preserved in RNAlater (Qiagen) for PCR or formalin-fixed for IHC.

### Immunohistochemistry

Paraffin-embedded formalin-fixed xenograft tumor samples were sectioned and stained with H&E (Sigma) to assess tumor morphology, as well as with select immunohistochemical antibodies. To determine resemblance to RMS, immunohistochemical analysis included anti-desmin, anti-MyoD, and anti-myogenin. Slides were evaluated by pathologists (R.C.B., E.R.) with experience in pediatric sarcomas. YAP IHC was performed as previously described [19]. TAZ IHC (Sigma #HPA007415), TUNEL (Trevigen #4810-30-K) and Ki67 (Dako #M7240) staining were performed per the manufacturer’s protocols. TAZ staining was scored on a scale of 0–4 by two blinded scorers (0 = no staining, 1 = <25% staining, 2 = 25–50% staining, 3 = 50–75% staining, 4 = >75% staining). Four images were scored per tumor and averaged. TUNEL and Ki67 slides were photographed, positively and negatively stained cells were counted manually with the aid of cell counting software (ImageJ, NIH), and five images were counted per condition.

### Drug Studies

Verteporfin was obtained from Proactive Molecular Research P17-0440 and was dissolved in DMSO at 100mg/ml. *In vitro*, verteporfin was diluted to 10μM in cell culture media. For *in vivo* experiments, verteporfin was diluted to 10mg/ml in PBS and administered by intraperitoneal injection at 100mg/kg every other day for eight treatments total.

### Statistical Analysis

Statistical analysis was performed using GraphPad Prism (GraphPad). Unless otherwise noted, data is presented as the mean and SE. One-way ANOVA, two-way ANOVA, and unpaired T-test were used as appropriate. P values were considered significant at *, P< 0.05; **, P<0.01; ***, P<0.001; and ****, P<0.0001.

## Results

### YAP expression is required for human Ras-driven RMS cell growth and supports proliferation and survival *in vitro*

**Fig 1:**
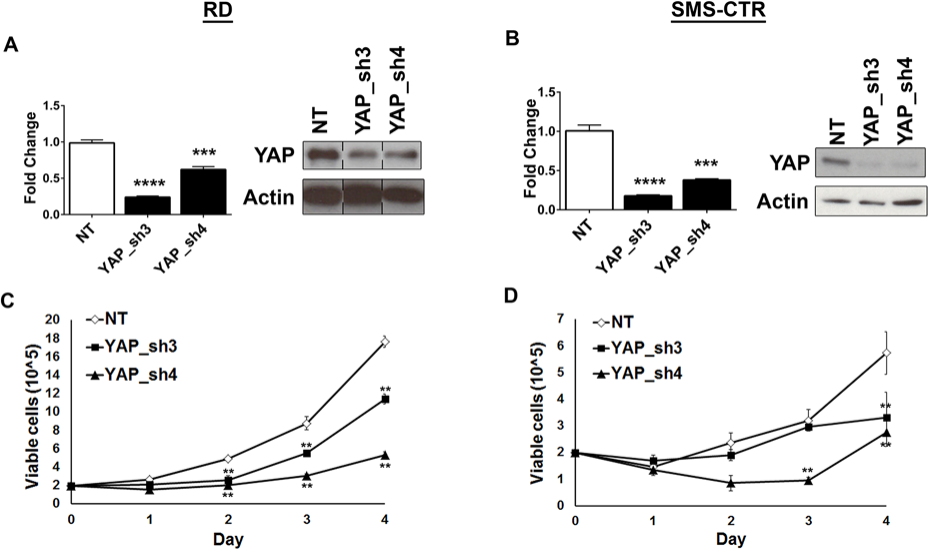
YAP suppression inhibits eRMS cell growth. **(A)** YAP suppression by shRNA in RD cells is validated by qRT-PCR (left) and immunoblot (right). Lanes in the immunoblot are from the same membrane but have been rearranged into this order. **(B)** YAP suppression validation in SMS-CTR cells by qRT-PCR (left) and immunoblot (right). YAP suppression in **(C)** RD and **(D)** SMS-CTR cells inhibits cell growth as measured by manual cell counting over four days. **, P<0.01; ***, P<0.001; and ****, P<0.0001.

**Fig 2:**
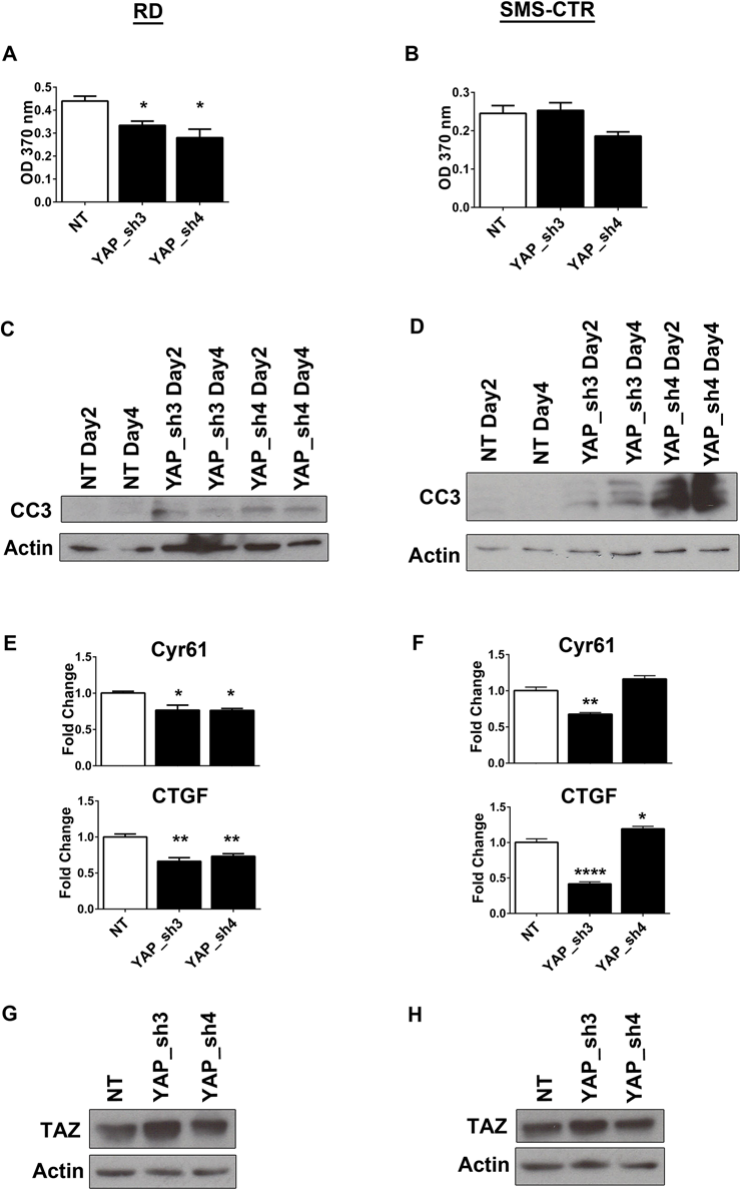
YAP suppression inhibits proliferation and stimulates apoptosis. Cell proliferation, as measured by BrdU incorporation, is decreased in **(A)** RD and **(B)** SMS-CTR cells stably expressing YAP shRNAs. Immunoblot for cleaved caspase 3 (CC3) shows an increase in apoptosis at days 2 and 4 after shRNA expression in **(C)** RD and **(D)** SMS-CTR cells. qRT-PCR for YAP target genes *Cyr61* and *CTGF* show decreased expression with YAP suppression in **(E)** RD and **(F)** SMS-CTR cells. Immunoblot analysis of TAZ in **(G)** RD and **(H)** SMS-CTR cells expressing YAP shRNAs. *, P< 0.05; **, P<0.01; and ****, P<0.0001.

To assess the role of the YAP oncoprotein in human eRMS, YAP was suppressed in both RD (*NRAS* mutant) [25] and SMS-CTR (*HRAS* mutant) [26] human eRMS cell lines using lentiviral-mediated shRNA expression. Two independently-targeting shRNAs, which suppressed YAP at the mRNA and protein levels in both RD and SMS-CTR cells **(Fig 1A and 1B),** inhibited cell growth as measured by cell counting over time **(Fig 1C and 1D)** which is in alignment with recent studies in RD cells [17]. However, since growth curves yield information only about overall population growth, further studies were done to determine the mechanism of growth inhibition. YAP deficiency was found to interfere with RD and SMS-CTR cell proliferation, as measured by BrdU incorporation **(Fig 2A and 2B)**, and stimulate apoptosis, as measured by cleaved caspase 3 expression **(Fig 2C and 2D)**. Since *Cyr61* (cysteine-rich, angiogenic inducer, 61) and *CTGF* (connective tissue growth factor) are established transcriptional targets of YAP, the expression of these genes was measured in both RD and SMS-CTR cells in the setting of the shRNAs using quantitative real-time PCR (qRT-PCR), and indeed their decreased expression correlated with YAP suppression **(Fig 2E)** in RD cells. In SMS-CTR cells, YAP_sh3 induced the expected decrease in *CTGF* and *Cyr61* while YAP_sh4 showed no change **(Fig 2F)**. *CTGF* expression has previously been associated with senescent cells and the possibility that YAP_sh4 is causing cellular senescence should be explored [33]. Thus, *in vitro*, the YAP oncoprotein promotes cell proliferation and survival in Ras-driven eRMS. As recent work has shown that YAP suppression results in an upregulation of TAZ protein levels [34], we investigated the levels of TAZ protein after YAP suppression. In both RD **(Fig 2G)** and SMS-CTR **(Fig 2H)** cells, TAZ protein levels increased upon expression of the YAP shRNAs. Since YAP suppression did not completely ablate cell growth **(Fig 1C and 1D)**, we hypothesize the upregulation of TAZ may partially compensate for the loss of YAP in these cells.

### YAP suppression delays tumor growth *in vivo*

**Fig 3:**
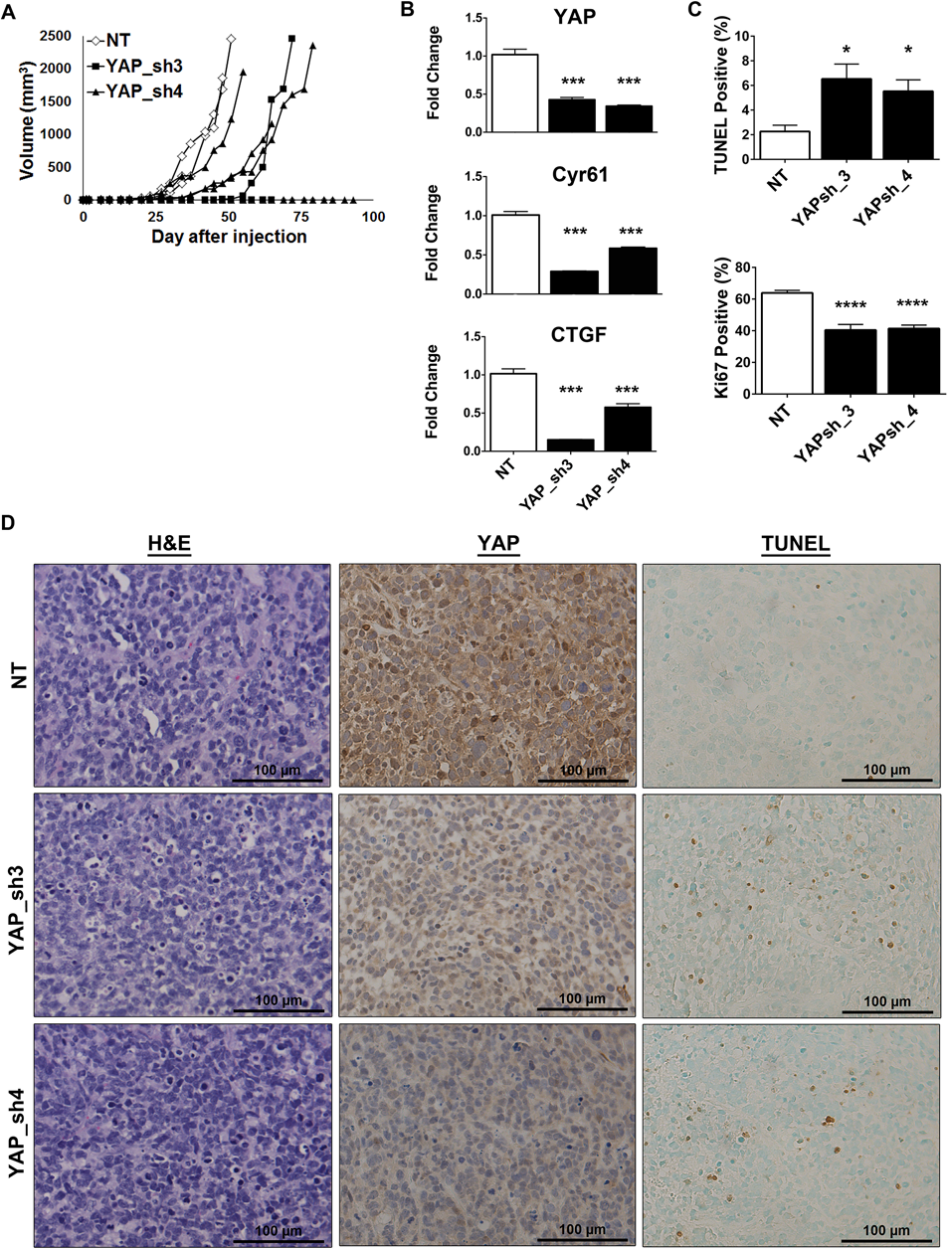
YAP suppression delays tumor growth *in vivo*. **(A)** *In vivo* YAP suppression in SMS-CTR xenografts inhibits tumor growth over time. (◊, NT; ■, YAP_sh3; ▲, YAP_sh4). **(B)** qRT-PCR for *YAP*, *Cyr61*, and *CTGF* show decreased expression in SMS-CTR xenografts expressing YAP shRNAs. Bars represent the average of each treatment group. **(C)** Quantitation of TUNEL (top) and Ki67 (bottom) staining reflects an increase in apoptosis and decrease in cell proliferation, respectively. **(D)** Representative images of H&E (left), YAP IHC (middle) and TUNEL staining (right) of the xenograft tumors. *, P<0.05; ***, P<0.001; and ****, P<0.0001. Scale bars: 100μm.

To explore the role of YAP suppression in RMS tumorigenesis *in vivo*, we established subcutaneous xenografts of SMS-CTR (*HRAS*-mutant) cells stably expressing the YAP shRNAs, or a non-targeting (NT) control. Previous work showed that YAP inhibition in RD (*NRA*S-mutant) xenografts decreased tumor burden [17]. We found that expression of the YAP shRNAs in the SMS-CTR xenografts delayed tumor growth as compared to the NT control **(Fig 3A)**. We validated YAP suppression within the xenografts at both the mRNA **(Fig 3B)** and protein levels **(Fig 3D, middle)**. YAP suppression was maintained in the tumors and this suppression also resulted in decreased expression of the YAP target genes *Cyr61* and *CTGF* **(Fig 3B)**. We next examined the impact of the YAP shRNAs on tumor morphology. However, H&E analysis showed no obvious differences between the treatment groups **(Fig 3D, left)**. To determine the mechanism of tumor growth delay, TUNEL and Ki67 staining were performed to assess the levels of apoptosis and cell proliferation, respectively. As observed previously *in vitro* **(Fig 2D),** YAP suppression *in vivo* increased apoptosis **(Fig 3C, Fig 3D, right)**. Ki67 staining levels appeared visually similar but upon quantification there was a significant decrease in cell proliferation with expression of the YAP shRNAs. **(Fig 3C)**. Although tumors showed sustained *YAP*, *Cyr61*, and *CTGF* loss, they were still able to eventually grow to the IACUC-defined tumor burden, suggesting that RMS cells have mechanisms to overcome YAP inhibition, and this may be due to the activity of oncogenic Ras or other compensatory pathways. Because *in vitro* expression of the YAP shRNAs induced upregulation of TAZ protein **(Fig 2G and 2H)**, we investigated the levels of TAZ protein in the tumors by IHC **(S1 Fig)**. Although there were subtle changes in TAZ expression, due to tumor variability there was not a significant difference upon quantitation **(S1 Fig)**. Interestingly, some of the shRNA-expressing cells did not implant to form tumors. This may be due to technical reasons, or YAP suppression may alter the cells’ ability to initiate tumorigenesis and this should be explored in future studies. These experiments suggest that YAP oncoprotein expression is important for Ras-driven eRMS tumor growth.

### Pharmacologic inhibition of YAP

**Fig 4:**
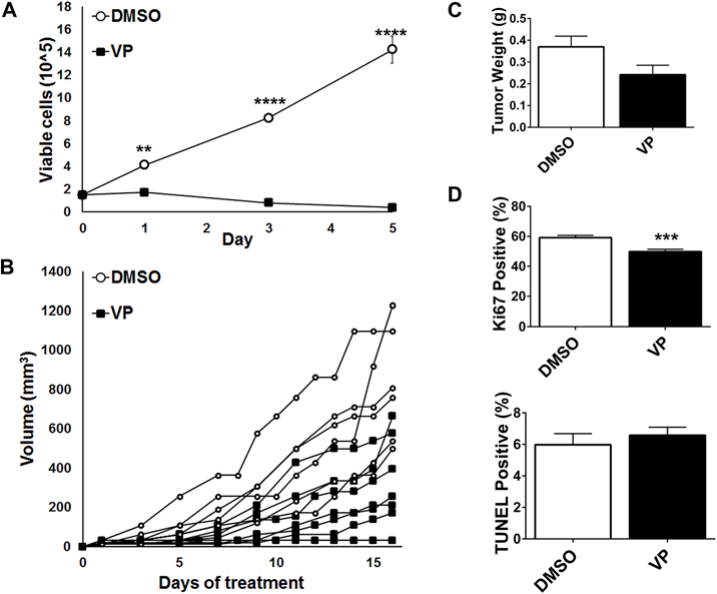
Pharmacologic inhibition of YAP inhibits tumor growth *in vivo*. **(A)** SMS-CTR cells treated with 10μM VP have decreased cell growth, measured by manual cell counting over five days. **(B)** SMS-CTR subcutaneous xenografts treated with 100mg/kg VP have decreased tumor growth as compared to vehicle control (DMSO). **(C)** The average tumor weight of VP treated mice is decreased compared to control. **(D)** Ki67 staining is decreased in VP-treated mice, but TUNEL staining remains the same. **, P<0.01; ***, P<0.001; and ****, P<0.0001. Scale bars: 100μm.

Since genetic suppression of YAP inhibited eRMS cell growth *in vitro* and *in vivo*, we next examined the effect of pharmacologic inhibition of YAP on Ras-driven RMS. Verteporfin (VP) is an FDA approved drug for treatment of macular degeneration that was recently determined to also be an inhibitor of YAP-TEAD interactions [35]. VP can reverse YAP-driven liver overgrowth *in vivo* [35], and inhibit cancer cell growth *in vitro* and *in vivo* [36–39]. In SMS-CTR cells, 10μM VP abrogated cell growth over five days **(Fig 4A)**. Since there was a profound effect *in vitro*, we evaluated the effect of VP in a murine subcutaneous xenograft model. Once tumors were palpable, mice were treated with VP or DMSO (vehicle) every other day for eight doses. In most animals, VP inhibited tumor growth and resulted in decreased tumor weight **(Fig 4B and 4C).** However, there was variability in the responses and two tumors from the VP-treated animals grew at the same rate as the vehicle control. Cell proliferation, as measured by Ki67 staining, was significantly decreased with VP treatment **(Fig 4D**), while apoptosis measured by TUNEL staining was not significantly changed. Surprisingly, VP treatment did not change the levels of YAP target genes *Cyr61* and *CTGF* **(S2 Fig)**.

### YAP promotes an undifferentiated state

**Fig 5:**
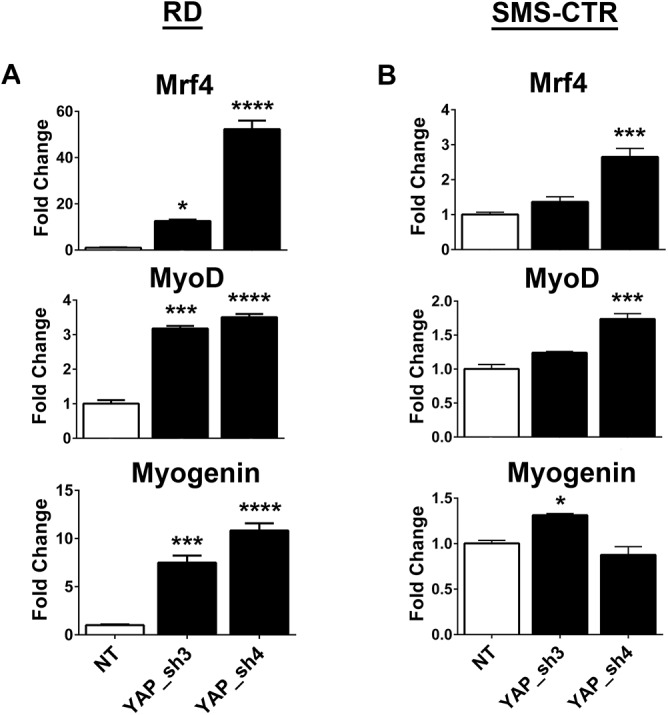
YAP suppression promotes myogenic transcription factor expression. qRT-PCR for myogenic differentiation genes *Mrf4*, *MyoD*, and *Myogenin* show increased expression with YAP suppression in **(A)** RD and **(B)** SMS-CTR cells. *, P< 0.05; ***, P<0.001; and ****, P<0.0001.

As a malignancy associated with the skeletal muscle lineage, RMS pathogenesis includes deficiencies in the ability of cells to terminally differentiate (summarized in [40]). Therefore, cellular programs must exist to block differentiation. To determine whether YAP participates in this program, eRMS cells were examined for expression of myogenic markers after YAP-targeted shRNA suppression. shRNA-suppressed cells had a 3–60 fold increase in myogenic differentiation markers *Mrf4*, *MyoD* and *myogenin* **(Fig 5A**), suggesting that indeed YAP expression contributes to the block in terminal differentiation. Similar results were observed in the SMS-CTR cells **(Fig 5B)**.

**Fig 6:**
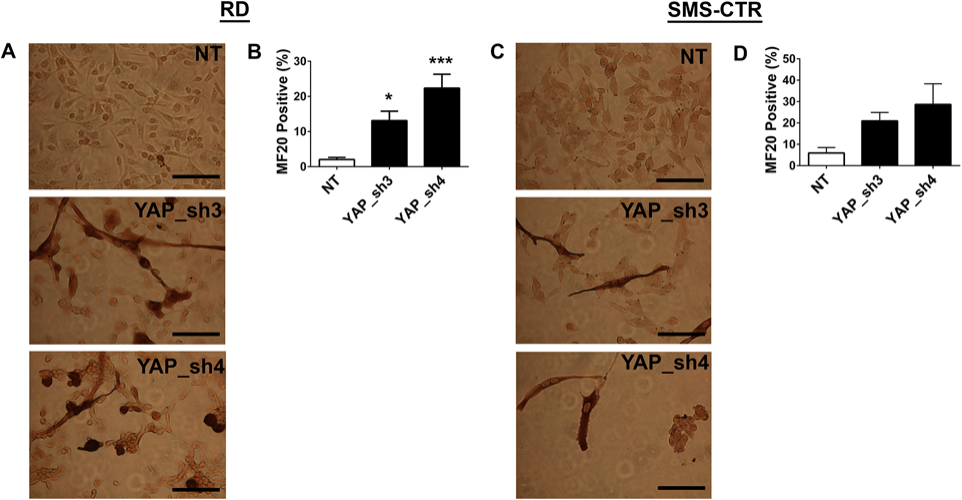
eRMS cell lines show increased differentiation with YAP knockdown. Cells stably expressing YAP shRNAs were cultured in differentiation media for five days, then stained for MF20 expression. Representative images and quantitation of MF20 staining in **(A, B)** RD and **(C, D)** SMS-CTR cells. *, P< 0.05; ***, P<0.001. Scale bars: 62.5μm.

When YAP shRNA-suppressed RD and SMS-CTR cells were cultured in differentiation media, their growth slowed markedly (as opposed to cells expressing control vector, which continued to proliferate), and cells changed to an elongated morphology **(Fig 6A and 6C)**. A concomitant increase in the number of cells staining positive for MF20 (which stains sarcomeric myosin) was evident **(Fig 6A and 6C)**, and this was found to be statistically significant when quantified **(Fig 6B and 6D)**. This data is consistent with the previously identified role for YAP in murine eRMS tumors, murine muscle satellite cells, and C2C12 murine myoblasts [17, 41, 42]. Thus, in concert with these prior studies, YAP functions to block differentiation in skeletal muscle cells and Ras-mutant eRMS tumors, and differentiation therapy could be a potential therapeutic approach for this cancer.

### YAP expression enables bypass of the senescence checkpoint

**Fig 7:**
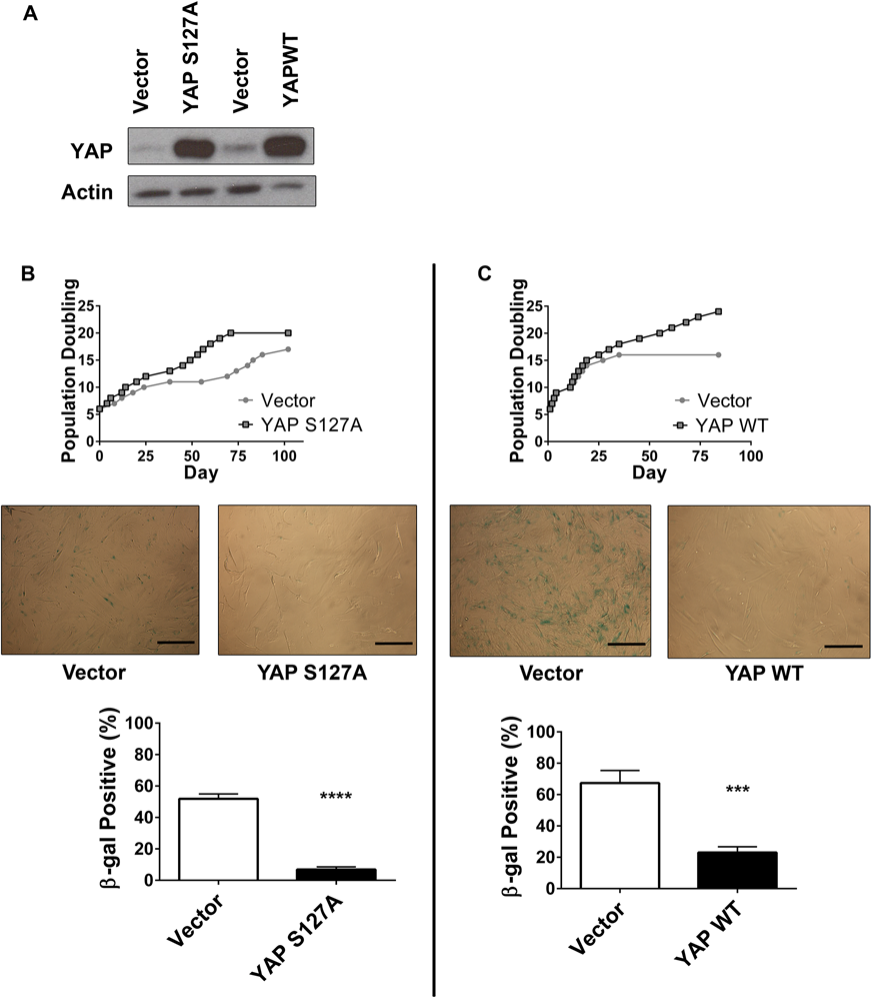
YAP expression enables senescence bypass. **(A)** Total YAP protein levels in HSMMs ectopically expressing vector, YAPS127A, or YAPWT. **(B)** Top, population doublings over time of HSMMs expressing vector (●) or YAPS127A (■). Middle, vector-expressing cells show increased β-gal staining as compared to YAPS127A. Bottom, quantitation of β-gal staining. **(C)** Population doublings of HSMMs expressing YAPWT (top) and β-gal staining (bottom, middle). β-gal staining was performed at PD 16 (Vectors, YAPS127A) and PD 18 (YAPWT). ***, P<0.001; ****, P<0.0001. Scale bars: 125μm.

Genetic modeling provides insight into the phenotypic contributions of gain-of-function of oncogenes and loss-of-function of tumor suppressors. Thus, we examined the role of YAP by substituting it into our previously established genetically-defined model of Ras-mutant eRMS. (As discussed this model relies on serial stable expression of the *SV40* early region, *hTERT*, and oncogenic *HRASG12V*.) Since telomere maintenance and Ras expression are obligatory steps in the tumorigenic process of Ras-driven eRMS [8], we reasoned that YAP would substitute for the SV40 early region in our model, and provide the stimulus to enable continuous growth in culture. Therefore, we stably expressed either YAPS127A or wild-type YAP (YAPWT) in primary human myoblasts **(Fig 7A)**, and monitored cells for population doubling and cellular morphology. Although YAP gain-of-function point mutations have not been reported in human RMS samples, increases in YAP copy number have been described, providing rationale to study both the mutant and wild type YAP constructs [17]. As previously observed [32], HSMMs expressing a control vector ceased proliferating after 10–15 population doublings (PD), having reached a tissue culture-induced senescence. However, cells expressing YAPS127A continued proliferating past this checkpoint **(Fig 7B).** Onset of senescence in vector cells was verified by acquisition of β-galactosidase (β-gal) expression, a biochemical marker of senescence (blue color and quantitation shown in **Fig 7B middle, bottom**), while cells expressing YAPS127A were β-gal negative. Similarly, cells expressing YAPWT bypassed senescence in culture and remained β-gal negative **(Fig 7C)**. These data suggest that YAP expression provides cellular signals to dampen or ignore pro-senescence stimuli.

### YAP primes myoblasts for oncogenic Ras-driven RMS tumorigenesis *in vivo*

**Fig 8:**
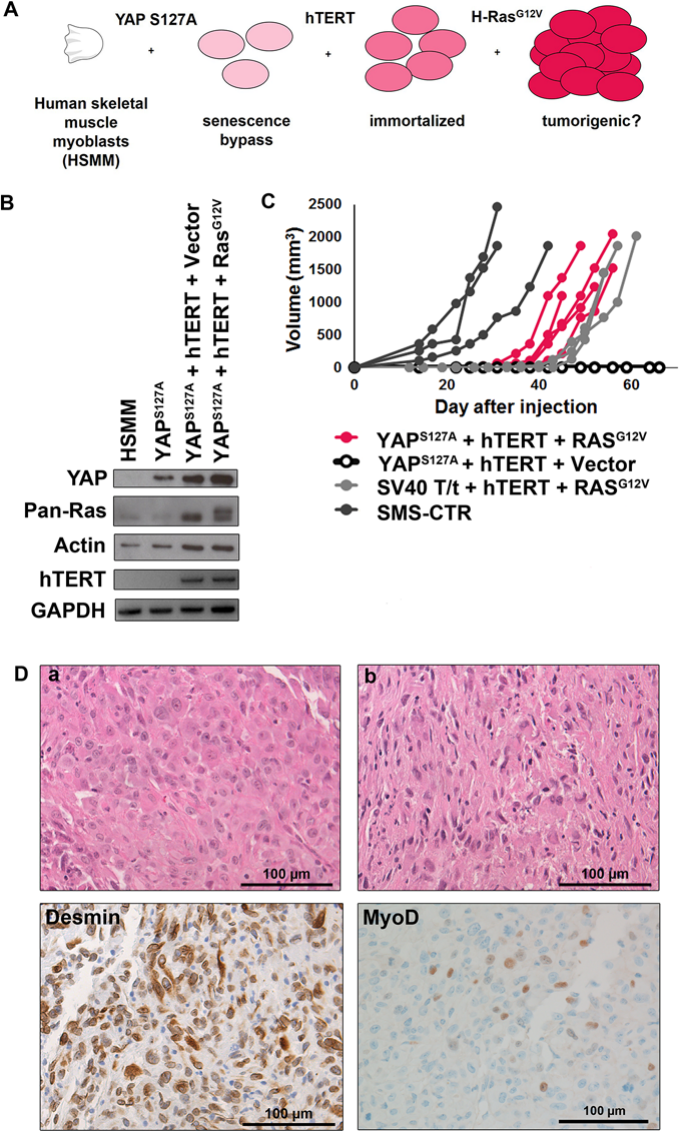
A genetically defined model of RMS based on serial stable expression of YAP, hTERT and oncogenic Ras. **(A)** Schematic of the “YHR” genetic model predicted to form xenograft tumors. **(B)** Immunoblot validation of YAP, Ras, and actin expression (top) and RT-PCR validation of hTERT and GAPDH expression (bottom) in cell lines. In the pan-Ras blot the top band is the epitope (FLAG)-tagged exogenous oncogenic Ras and the bottom band is the endogenous Ras. **(C)** Tumor volume as measured over time of YHR (pink), THR (light gray, historical data), SMS-CTR (dark gray) and YHV (black with open circles) xenografts. **(D)** H&E of two individual YHR tumors (a, b) and representative immunohistochemistry of desmin and MyoD of YHR tumors to confirm skeletal muscle markers (bottom). Scale bars: 100μm.

Since the stable expression of YAP permitted myoblasts to proliferate past the senescence checkpoint, we assessed whether completing the model by adding hTERT and subsequently oncogenic HRasG12V, abbreviated “YHR” (YAPS127A-hTERT-HRasG12V), would lead to a tumorigenic phenotype **(Fig 8A)**. We also generated the accompanying control cell line, “YHV” (YAPS127A-hTERT-Vector). Previous work demonstrated that “VHR” (Vector-hTERT-HRasG12V) cells are not tumorigenic as the senescence checkpoint remains intact and oncogenic Ras is not tolerated, leading to cell senescence followed by cell death [8]. Expression of transgenes in the cell lines was verified by PCR or immunoblot **(Fig 8B)**. Based on the recent GEMM work showing that YAPS127A expression in activated satellite cells is sufficient for eRMS tumorigenesis [17], we predicted that both YHV and YHR cell lines would generate tumor xenografts in immunodeficient mice. However, we found that compared to the internal positive control cell line (SMS-CTR) and our previous model (THR), YHR but not YHV cells were tumorigenic, and the YHR tumors formed with 100% penetrance **(Fig 8C)**. To address the possibility that we had not waited long enough for tumor formation, an additional cohort of mice were injected with YHV cells and have also not formed tumors in over 150 days (data not shown). This suggests that in our human primary cell-based model of Ras-driven RMS, YAPS127A alone was not sufficient to initiate and support tumorigenesis. To determine if a different oncogene could substitute for Ras in our model, we created cells expressing YAPS127A, *hTERT*, and *MYCN*. *MYCN* was expressed in the same founder population of YAPS127A + hTERT cells and injected subcutaneously with the same lot of Matrigel. However, *MYCN* was not sufficient for tumorigenesis **(S3 Fig)**. This suggests that cooperation between YAP and Ras signaling specifically initiates tumorigenesis in this model.

H&E analysis of the YHR tumors revealed a spectrum of morphologic patterns **(Fig 8D,a-b)**, which is also observed in human clinical RMS samples (summarized in [2]). The tumors were all high-grade sarcomas with abundant necrosis and a high proliferative rate. Most were characterized by spindled to epithelioid cells with large, pleomorphic nuclei and prominent nucleoli consistent with pRMS **(Fig 8D,a)**. One tumor was composed of diffuse spindled cells and smaller nuclei consistent with eRMS with anaplasia **(Fig 8D,b)**. Within each tumor, rare cells had multiple central nuclei and elongated tails of eosinophilic cytoplasm suggesting rhabdomyoblastic differentiation, although definite cross striations were not identified. Staining with desmin and MyoD, markers used clinically in evaluating soft tissue sarcomas, validated the skeletal muscle phenotype required for the assignment of RMS **(Fig 8D, bottom)**. All tumors showed uniform desmin expression and scattered MyoD1 positive nuclei but were negative for myogenin. Although myogenin staining is associated with an RMS diagnosis, only about 50% of pRMS stain positively for myogenin and some eRMS can be negative or have very low levels of myogenin [43, 44]. Therefore, the YHR model tumors are histologically consistent with human Ras-driven RMS.

## Discussion

The Hippo pathway is a tumor suppressor network shown to be dysregulated in many adult epithelial solid tumors including ovarian, lung, and liver [45–47]. Recent work has also shown a role for YAP in sarcomas, tumors of mesenchymal origin, and in skeletal muscle cells [17, 19, 41, 42, 48]. Here we investigate the role of YAP in Ras-driven RMS. YAP-directed shRNAs inhibited human Ras-driven RMS cell proliferation, promoted apoptosis, and stimulated myogenic differentiation *in vitro*, as well as delayed tumor growth *in vivo*. Pharmacologic inhibition of YAP with verteporfin also decreased cell growth *in vitro* and *in vivo*. In our human primary cell-based model, YAP expression was sufficient to support myoblasts through the senescence checkpoint, priming them for subsequent oncogenic Ras expression, resulting in a fully transformed cell that generates tumors in a murine xenograft system. The resulting YAP and Ras-driven tumors show a range of morphologies, consistent with human RMS. Together these studies elucidate the importance of YAP in tumor initiation and maintenance in Ras-driven RMS.

Previous work has identified a connection between YAP and Ras in mammalian epithelial tumorigenesis [49, 50]. For example, in murine models of pancreatic and lung cancers, YAP can replace oncogenic Kras as a tumorigenic driver during Ras-independent tumor recurrence [49, 50]. However, in our human cell-based model of RMS both YAP and Ras are required, as YAP expression alone does not initiate tumor formation. This observation is relevant for therapeutic planning, as inhibition of Ras or YAP signaling alone may not be sufficient, suggesting that combination therapy should be explored to inhibit both pathways simultaneously [51]. In our study, VP perturbed but did not ablate tumor growth, so combining VP with other chemotherapies, such as Ras pathway inhibitors, may increase its efficacy. We found VP to have limited solubility, and variable deposits of VP were observed in the intraperitoneal space of the mice. This suggests that the mice were receiving an inconsistent dose and a different formulation of VP to increase solubility may be required, as others have observed [52]. Additionally, although VP decreased tumor cell proliferation, there was not a significant effect on the transcription of YAP target genes *Cyr61* and *CTGF* **(S2 Fig).** This suggests that VP is not specific to YAP-TEAD interactions and may have other effects *in vivo*, which should be considered in future studies.

The current studies provide insight into the temporal function of YAP in RMS tumorigenesis. In our previous “THR” model, expression of the *SV40* early region in human myoblasts enabled senescence bypass by inhibiting Rb and p53, known enforcers of cellular senescence [8]. In the current YHR model, YAP must therefore be functioning (either directly or indirectly) to inhibit these pathways. Previous work suggested a link between YAP and senescence through YAP’s transcriptional regulation of the cyclin-dependent kinase CDK6. When fibroblasts reach culture-induced senescence YAP levels decrease; conversely, YAP knockdown can induce premature senescence by downregulation of CDK6 [53]. It remains to be seen whether this circuit is active in our system. Our work demonstrates that YAP activation may be an early event in Ras-driven RMS tumorigenesis, and this early event permits the subsequent expression of oncogenic Ras.

Recent GEMM models also suggest an important role for YAP signaling in RMS [17]. Conditional expression of YAPS127A in activated (through cardiotoxin-mediated injury) murine skeletal muscle satellite cells was sufficient for eRMS tumorigenesis. However, YAPS127A expression itself did not activate these satellite cells, and given their quiescence they did not alter muscle morphology or induce tumorigenesis. These results suggest that hyperactive YAP signaling and satellite cell activation cooperate in tumorigenesis. Surprisingly, our work in proliferating HSMMs suggests that hyperactive YAP signaling is not sufficient for tumorigenesis. That is, HSMMs expressing YAPS127A in combination with hTERT (hTERT is used in human tumor modeling, but not needed in GEMMs since mice have very long telomeres [8, 54–56]) were not able to form tumors in xenograft models. The final step, expression of an oncogenic Ras mutant, was required. Intriguingly, this mirrors our original model of eRMS, which also requires oncogenic Ras signaling as a final tumorigenic step [8]. Alternative possibilities are that the level of YAP activation may have been insufficient for tumorigenesis, or perhaps in HSMMs there is no level of YAP activity that is tumorigenic without additional oncogenes. Understanding the role of oncogenic Ras in this context, and how it provides pro-tumorigenic signals that can replace those found in activated satellite cells, will be an important aspect of future work.

Last, this study raises questions about the role of oncogenic Ras mutations in RMS tumor morphology. While the recently published GEMM of RMS based on expression of YAPS127A in activated satellite cells yielded eRMS [17], both an oncogenic Kras-driven GEMM of RMS [9] and the current cell-based model resulted in a spectrum of tumor morphologies, from embryonal to pleomorphic RMS. This may be due to the inherent biology of oncogenic Ras and how it functions in skeletal muscle to drive RMS tumorigenesis, suggesting that Ras-driven RMS tumors are a distinct subgroup with a range of morphologies and a worse prognosis [13]. Additional studies of the role of Ras-driven RMS tumorigenesis—and its relationship to YAP—are necessary to understand the mechanism of tumorigenesis and to develop appropriate therapies for these aggressive tumors.

In summary, the YAP oncoprotein functions to support proliferation, survival, an undifferentiated state, and *in vivo* tumorigenesis of human Ras-driven RMS cell lines, thus contributing to the maintenance of the tumorigenic phenotype. Using a primary human myoblast-based model, oncogenic YAP also serves as an initial genetic lesion, permitting bypass of senescence and priming myoblasts to tolerate subsequent expression of hTERT and oncogenic Ras, which are necessary and sufficient to form xenograft tumors *in vivo*. This work provides a novel tool to explore how YAP and Ras function in human cells during RMS tumorigenesis, and lays the groundwork for future preclinical investigations of these essential pathways.

## Supporting Information

S1 Fig TAZ expression in tumors with YAP suppression. **(A)** Representative images of TAZ IHC on NT, YAP_sh3, and YAP_sh4 tumors. **(B)** Quantitation of TAZ IHC. Tumors were scored on a scale of 0–4, four images were scored per tumor and scores averaged. There was not a significant difference between the groups. Scale bars: 100μm.
(TIF)

S2 Fig YAP target gene expression in VP-treated tumors. **(A)** qRT-PCR of *Cyr61* and *CTGF* do not change with VP treatment.
(TIF)

S3 Fig YAPS127A +hTERT +MycN expressing cells do not form tumors. **(A)** Validation of expression of all oncogenes by immunoblot (YAP, actin) or RT-PCR (hTERT, MycN, GAPDH). **(B)** None of the mice formed tumors.
(TIF)

S1 Table Primers for semi-quantitative and quantitative PCR. (PDF)

## References

[1] Ognjanovic S, Linabery AM, Charbonneau B, Ross JA. Trends in childhood rhabdomyosarcoma incidence and survival in the United States, 1975–2005. Cancer. 2009;115(18):4218–26. 10.1002/cncr.24465 19536876 PMC2953716

[2] Egas-Bejar D, Huh WW. Rhabdomyosarcoma in adolescent and young adult patients: current perspectives. Adolescent health, medicine and therapeutics. 2014;5:115–25. 10.2147/AHMT.S44582 24966711 PMC4069040

[3] Perkins SM, Shinohara ET, DeWees T, Frangoul H. Outcome for children with metastatic solid tumors over the last four decades. PLoS One. 2014;9(7):e100396 10.1371/journal.pone.0100396 25003594 PMC4086810

[4] Stratton MR, Fisher C, Gusterson BA, Cooper CS. Detection of point mutations in N-ras and K-ras genes of human embryonal rhabdomyosarcomas using oligonucleotide probes and the polymerase chain reaction. Cancer research. 1989;49(22):6324–7. 2680062

[5] Wilke W, Maillet M, Robinson R. H-ras-1 point mutations in soft tissue sarcomas. Modern pathology: an official journal of the United States and Canadian Academy of Pathology, Inc. 1993;6(2):129–32. 8483882

[6] Chen Y, Takita J, Hiwatari M, Igarashi T, Hanada R, Kikuchi A, et al Mutations of the PTPN11 and RAS genes in rhabdomyosarcoma and pediatric hematological malignancies. Genes, chromosomes & cancer. 2006;45(6):583–91. 16518851 10.1002/gcc.20322

[7] Hahn WC, Counter CM, Lundberg AS, Beijersbergen RL, Brooks MW, Weinberg RA. Creation of human tumour cells with defined genetic elements. Nature. 1999;400(6743):464–8. 10440377 10.1038/22780

[8] Linardic CM, Downie DL, Qualman S, Bentley RC, Counter CM. Genetic modeling of human rhabdomyosarcoma. Cancer research. 2005;65(11):4490–5. 15930263 10.1158/0008-5472.CAN-04-3194

[9] Blum JM, Ano L, Li Z, Van Mater D, Bennett BD, Sachdeva M, et al Distinct and overlapping sarcoma subtypes initiated from muscle stem and progenitor cells. Cell reports. 2013;5(4):933–40. 10.1016/j.celrep.2013.10.020 24239359 PMC3893104

[10] Langenau DM, Keefe MD, Storer NY, Guyon JR, Kutok JL, Le X, et al Effects of RAS on the genesis of embryonal rhabdomyosarcoma. Genes & development. 2007;21(11):1382–95. 17510286 10.1101/gad.1545007 PMC1877750

[11] Hettmer S, Liu J, Miller CM, Lindsay MC, Sparks CA, Guertin DA, et al Sarcomas induced in discrete subsets of prospectively isolated skeletal muscle cells. Proceedings of the National Academy of Sciences of the United States of America. 2011;108(50):20002–7. 10.1073/pnas.1111733108 22135462 PMC3250188

[12] Tsumura H, Yoshida T, Saito H, Imanaka-Yoshida K, Suzuki N. Cooperation of oncogenic K-ras and p53 deficiency in pleomorphic rhabdomyosarcoma development in adult mice. Oncogene. 2006;25(59):7673–9. 16785989 10.1038/sj.onc.1209749

[13] Chen X, Stewart E, Shelat AA, Qu C, Bahrami A, Hatley M, et al Targeting oxidative stress in embryonal rhabdomyosarcoma. Cancer cell. 2013;24(6):710–24. 10.1016/j.ccr.2013.11.002 24332040 PMC3904731

[14] Shern JF, Chen L, Chmielecki J, Wei JS, Patidar R, Rosenberg M, et al Comprehensive genomic analysis of rhabdomyosarcoma reveals a landscape of alterations affecting a common genetic axis in fusion-positive and fusion-negative tumors. Cancer discovery. 2014;4(2):216–31. 10.1158/2159-8290.CD-13-0639 24436047 PMC4462130

[15] Zhao B, Tumaneng K, Guan KL. The Hippo pathway in organ size control, tissue regeneration and stem cell self-renewal. Nature cell biology. 2011;13(8):877–83. 10.1038/ncb2303 21808241 PMC3987945

[16] Harvey KF, Zhang X, Thomas DM. The Hippo pathway and human cancer. Nature reviews Cancer. 2013;13(4):246–57. 10.1038/nrc3458 23467301

[17] Tremblay AM, Missiaglia E, Galli GG, Hettmer S, Urcia R, Carrara M, et al The Hippo transducer YAP1 transforms activated satellite cells and is a potent effector of embryonal rhabdomyosarcoma formation. Cancer cell. 2014;26(2):273–87. 10.1016/j.ccr.2014.05.029 25087979

[18] Helias-Rodzewicz Z, Perot G, Chibon F, Ferreira C, Lagarde P, Terrier P, et al YAP1 and VGLL3, encoding two cofactors of TEAD transcription factors, are amplified and overexpressed in a subset of soft tissue sarcomas. Genes, chromosomes & cancer. 2010;49(12):1161–71. 10.1002/gcc.20825 20842732

[19] Crose LE, Galindo KA, Kephart JG, Chen C, Fitamant J, Bardeesy N, et al Alveolar rhabdomyosarcoma-associated PAX3-FOXO1 promotes tumorigenesis via Hippo pathway suppression. The Journal of clinical investigation. 2014;124(1):285–96. 10.1172/JCI67087 24334454 PMC3871220

[20] Hanahan D, Weinberg RA. Hallmarks of cancer: the next generation. Cell. 2011;144(5):646–74. 10.1016/j.cell.2011.02.013 21376230

[21] McAllister RM, Melnyk J, Finkelstein JZ, Adams EC Jr., Gardner MB. Cultivation in vitro of cells derived from a human rhabdomyosarcoma. Cancer. 1969;24(3):520–6. 4241949 10.1002/1097-0142(196909)24:3<520::aid-cncr2820240313>3.0.co;2-m

[22] Whang-Peng J, Knutsen T, Theil K, Horowitz ME, Triche T. Cytogenetic studies in subgroups of rhabdomyosarcoma. Genes, chromosomes & cancer. 1992;5(4):299–310. 1283318 10.1002/gcc.2870050405

[23] Naini S, Etheridge KT, Adam SJ, Qualman SJ, Bentley RC, Counter CM, et al Defining the cooperative genetic changes that temporally drive alveolar rhabdomyosarcoma. Cancer research. 2008;68(23):9583–8. 10.1158/0008-5472.CAN-07-6178 19047133 PMC2593800

[24] Hinson AR, Jones R, Crose LE, Belyea BC, Barr FG, Linardic CM. Human rhabdomyosarcoma cell lines for rhabdomyosarcoma research: utility and pitfalls. Frontiers in oncology. 2013;3:183 10.3389/fonc.2013.00183 23882450 PMC3713458

[25] Schaaf G, Hamdi M, Zwijnenburg D, Lakeman A, Geerts D, Versteeg R, et al Silencing of SPRY1 triggers complete regression of rhabdomyosarcoma tumors carrying a mutated RAS gene. Cancer research. 2010;70(2):762–71. 10.1158/0008-5472.CAN-09-2532 20068162

[26] Shukla N, Ameur N, Yilmaz I, Nafa K, Lau CY, Marchetti A, et al Oncogene mutation profiling of pediatric solid tumors reveals significant subsets of embryonal rhabdomyosarcoma and neuroblastoma with mutated genes in growth signaling pathways. Clinical cancer research: an official journal of the American Association for Cancer Research. 2012;18(3):748–57. 22142829 10.1158/1078-0432.CCR-11-2056 PMC3271129

[27] Zhang J, Smolen GA, Haber DA. Negative regulation of YAP by LATS1 underscores evolutionary conservation of the Drosophila Hippo pathway. Cancer research. 2008;68(8):2789–94. 10.1158/0008-5472.CAN-07-6205 18413746

[28] Xia Y, Zhang YL, Yu C, Chang T, Fan HY. YAP/TEAD co-activator regulated pluripotency and chemoresistance in ovarian cancer initiated cells. PLoS One. 2014;9(11):e109575 10.1371/journal.pone.0109575 25369529 PMC4219672

[29] Zhao B, Wei X, Li W, Udan RS, Yang Q, Kim J, et al Inactivation of YAP oncoprotein by the Hippo pathway is involved in cell contact inhibition and tissue growth control. Genes & development. 2007;21(21):2747–61. 17974916 10.1101/gad.1602907 PMC2045129

[30] Overholtzer M, Zhang J, Smolen GA, Muir B, Li W, Sgroi DC, et al Transforming properties of YAP, a candidate oncogene on the chromosome 11q22 amplicon. Proceedings of the National Academy of Sciences of the United States of America. 2006;103(33):12405–10. 16894141 10.1073/pnas.0605579103 PMC1533802

[31] Belyea BC, Naini S, Bentley RC, Linardic CM. Inhibition of the Notch-Hey1 axis blocks embryonal rhabdomyosarcoma tumorigenesis. Clinical cancer research: an official journal of the American Association for Cancer Research. 2011;17(23):7324–36. 21948088 10.1158/1078-0432.CCR-11-1004 PMC3241994

[32] Linardic CM, Naini S, Herndon JE 2nd, Kesserwan C, Qualman SJ, Counter CM. The PAX3-FKHR fusion gene of rhabdomyosarcoma cooperates with loss of p16INK4A to promote bypass of cellular senescence. Cancer research. 2007;67(14):6691–9. 17638879 10.1158/0008-5472.CAN-06-3210

[33] Kim KH, Park GT, Lim YB, Rue SW, Jung JC, Sonn JK, et al Expression of connective tissue growth factor, a biomarker in senescence of human diploid fibroblasts, is up-regulated by a transforming growth factor-beta-mediated signaling pathway. Biochemical and biophysical research communications. 2004;318(4):819–25. 15147944 10.1016/j.bbrc.2004.04.108

[34] Moroishi T, Park HW, Qin B, Chen Q, Meng Z, Plouffe SW, et al A YAP/TAZ-induced feedback mechanism regulates Hippo pathway homeostasis. Genes & development. 2015;29(12):1271–84. 10.1101/gad.262816.115 26109050 PMC4495398

[35] Liu-Chittenden Y, Huang B, Shim JS, Chen Q, Lee SJ, Anders RA, et al Genetic and pharmacological disruption of the TEAD-YAP complex suppresses the oncogenic activity of YAP. Genes & development. 2012;26(12):1300–5. 10.1101/gad.192856.112 22677547 PMC3387657

[36] Song S, Honjo S, Jin J, Chang SS, Scott AW, Chen Q, et al The Hippo Coactivator YAP1 Mediates EGFR Overexpression and Confers Chemoresistance in Esophageal Cancer. Clinical cancer research: an official journal of the American Association for Cancer Research. 2015;21(11):2580–90. 25739674 10.1158/1078-0432.CCR-14-2191 PMC4452384

[37] Ciamporcero E, Shen H, Ramakrishnan S, Yu Ku S, Chintala S, Shen L, et al YAP activation protects urothelial cell carcinoma from treatment-induced DNA damage. Oncogene. 2015 10.1038/onc.2015.219 26119935 PMC4695331

[38] Yu FX, Luo J, Mo JS, Liu G, Kim YC, Meng Z, et al Mutant Gq/11 promote uveal melanoma tumorigenesis by activating YAP. Cancer cell. 2014;25(6):822–30. 10.1016/j.ccr.2014.04.017 24882516 PMC4075337

[39] Brodowska K, Al-Moujahed A, Marmalidou A, Meyer Zu Horste M, Cichy J, Miller JW, et al The clinically used photosensitizer Verteporfin (VP) inhibits YAP-TEAD and human retinoblastoma cell growth in vitro without light activation. Experimental eye research. 2014;124:67–73. 10.1016/j.exer.2014.04.011 24837142 PMC4135181

[40] Keller C, Guttridge DC. Mechanisms of impaired differentiation in rhabdomyosarcoma. The FEBS journal. 2013;280(17):4323–34. 10.1111/febs.12421 23822136 PMC6250433

[41] Judson RN, Tremblay AM, Knopp P, White RB, Urcia R, De Bari C, et al The Hippo pathway member Yap plays a key role in influencing fate decisions in muscle satellite cells. Journal of cell science. 2012;125(Pt 24):6009–19. 10.1242/jcs.109546 23038772 PMC3585517

[42] Watt KI, Judson R, Medlow P, Reid K, Kurth TB, Burniston JG, et al Yap is a novel regulator of C2C12 myogenesis. Biochemical and biophysical research communications. 2010;393(4):619–24. 10.1016/j.bbrc.2010.02.034 20153295

[43] Yu L, Wang J. [Clinicopathologic features of pleomorphic rhabdomyosarcoma]. Zhonghua bing li xue za zhi Chinese journal of pathology. 2013;42(3):147–52. 10.3760/cma.j.issn.0529-5807.2013.03.002 23769431

[44] Dias P, Chen B, Dilday B, Palmer H, Hosoi H, Singh S, et al Strong immunostaining for myogenin in rhabdomyosarcoma is significantly associated with tumors of the alveolar subclass. The American journal of pathology. 2000;156(2):399–408. 10666368 10.1016/S0002-9440(10)64743-8 PMC1850049

[45] Fu D, Lv X, Hua G, He C, Dong J, Lele SM, et al YAP regulates cell proliferation, migration, and steroidogenesis in adult granulosa cell tumors. Endocrine-related cancer. 2014;21(2):297–310. 10.1530/ERC-13-0339 24389730 PMC4222524

[46] Lau AN, Curtis SJ, Fillmore CM, Rowbotham SP, Mohseni M, Wagner DE, et al Tumor-propagating cells and Yap/Taz activity contribute to lung tumor progression and metastasis. The EMBO journal. 2014;33(5):468–81. 10.1002/embj.201386082 24497554 PMC3989628

[47] Zhou D, Conrad C, Xia F, Park JS, Payer B, Yin Y, et al Mst1 and Mst2 maintain hepatocyte quiescence and suppress hepatocellular carcinoma development through inactivation of the Yap1 oncogene. Cancer cell. 2009;16(5):425–38. 10.1016/j.ccr.2009.09.026 19878874 PMC3023165

[48] Hsu JH, Lawlor ER. BMI-1 suppresses contact inhibition and stabilizes YAP in Ewing sarcoma. Oncogene. 2011;30(17):2077–85. 10.1038/onc.2010.571 21170084 PMC3276065

[49] Shao DD, Xue W, Krall EB, Bhutkar A, Piccioni F, Wang X, et al KRAS and YAP1 converge to regulate EMT and tumor survival. Cell. 2014;158(1):171–84. 10.1016/j.cell.2014.06.004 24954536 PMC4110062

[50] Kapoor A, Yao W, Ying H, Hua S, Liewen A, Wang Q, et al Yap1 activation enables bypass of oncogenic Kras addiction in pancreatic cancer. Cell. 2014;158(1):185–97. 10.1016/j.cell.2014.06.003 24954535 PMC4109295

[51] Lin L, Sabnis AJ, Chan E, Olivas V, Cade L, Pazarentzos E, et al The Hippo effector YAP promotes resistance to RAF- and MEK-targeted cancer therapies. Nature genetics. 2015;47(3):250–6. 10.1038/ng.3218 25665005 PMC4930244

[52] Yu FX, Zhang K, Guan KL. YAP as oncotarget in uveal melanoma. Oncoscience. 2014;1(7):480–1. 25594048 10.18632/oncoscience.57 PMC4278320

[53] Xie Q, Chen J, Feng H, Peng S, Adams U, Bai Y, et al YAP/TEAD-mediated transcription controls cellular senescence. Cancer research. 2013;73(12):3615–24. 10.1158/0008-5472.CAN-12-3793 23576552

[54] Kipling D. Telomere structure and telomerase expression during mouse development and tumorigenesis. Eur J Cancer. 1997;33(5):792–800. 9282119 10.1016/S0959-8049(97)00060-9

[55] Kim NW, Piatyszek MA, Prowse KR, Harley CB, West MD, Ho PL, et al Specific association of human telomerase activity with immortal cells and cancer. Science. 1994;266(5193):2011–5. 7605428 10.1126/science.7605428

[56] Shay JW, Bacchetti S. A survey of telomerase activity in human cancer. Eur J Cancer. 1997;33(5):787–91. 9282118 10.1016/S0959-8049(97)00062-2

