# Supplementary figures and images for: Role of the YAP Oncoprotein in Priming Ras-Driven Rhabdomyosarcoma
Source: PLoS One. 2015 Oct 23;10(10):e0140781. doi: 10.1371/journal.pone.0140781 (PMC4619859; doi:10.1371/journal.pone.0140781)

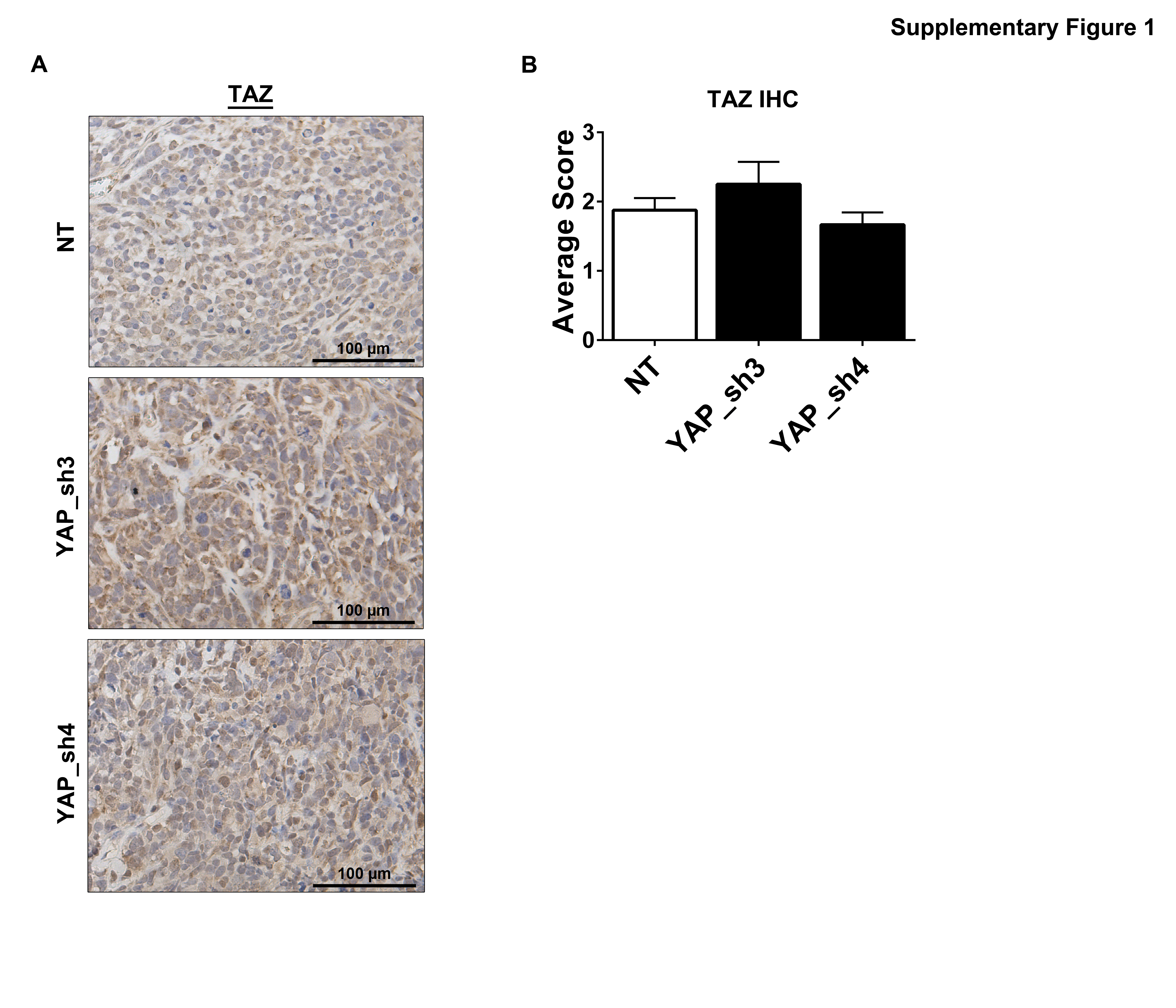

Supplement: S1 Fig — (A) Representative images of TAZ IHC on NT, YAP_sh3, and YAP_sh4 tumors. (B) Quantitation of TAZ IHC. Tumors were scored on a scale of 0–4, four images were scored per tumor and scores averaged. There was not a significant difference between the groups. Scale bars: 100μm. (TIF) [file pone.0140781.s001.tif]

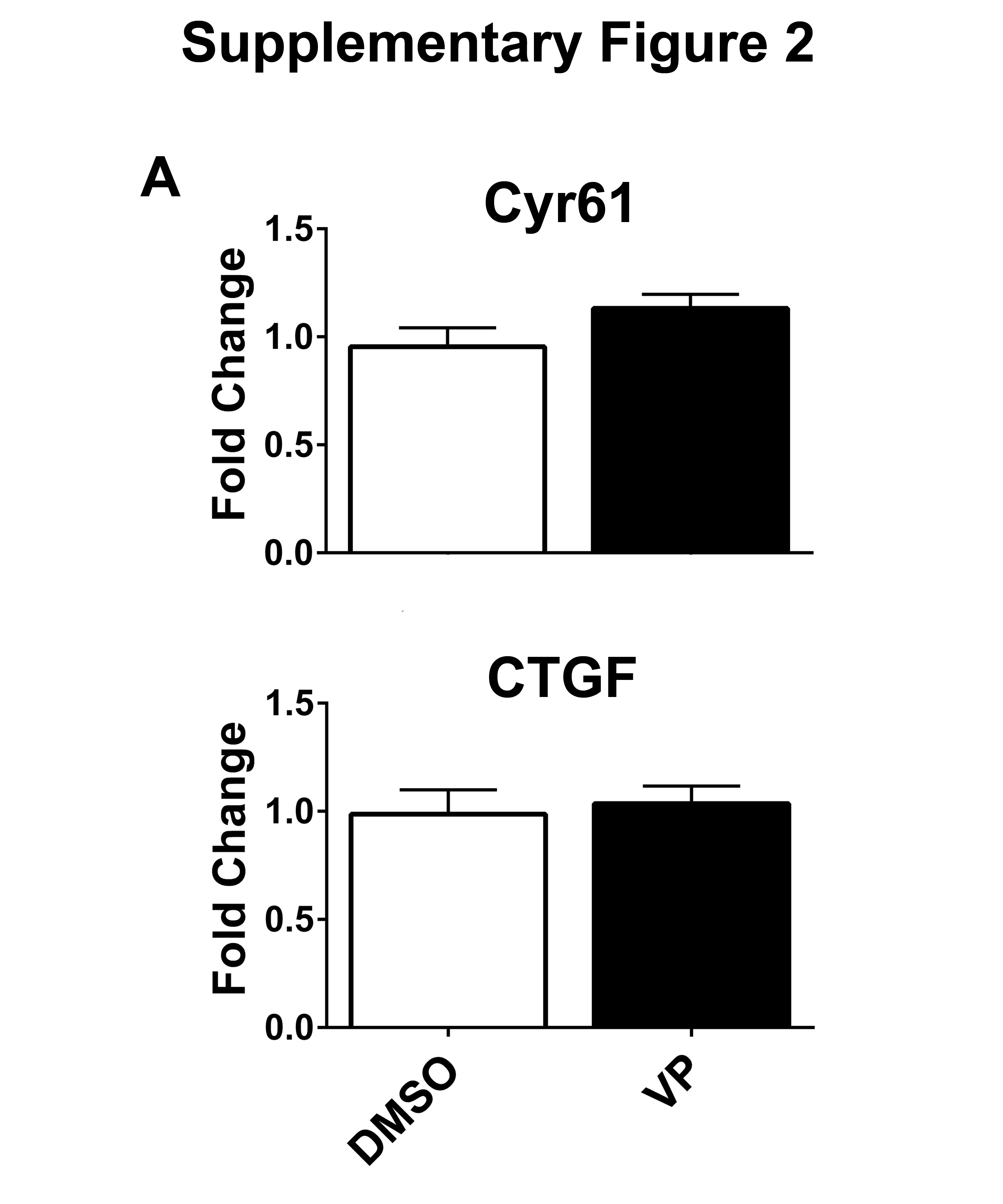

Supplement: S2 Fig — (A) qRT-PCR of Cyr61 and CTGF do not change with VP treatment. (TIF) [file pone.0140781.s002.tif]

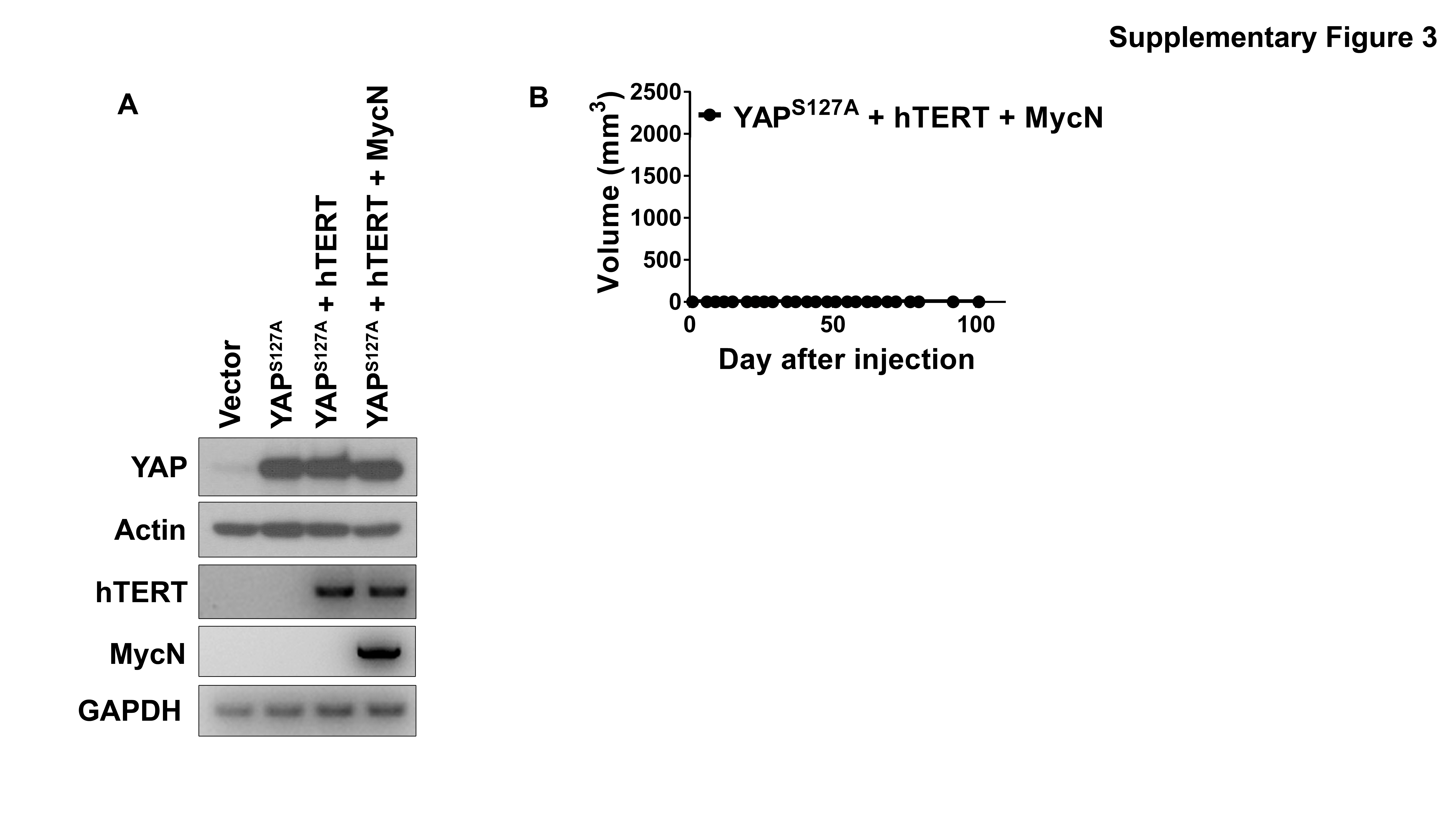

Supplement: S3 Fig — (A) Validation of expression of all oncogenes by immunoblot (YAP, actin) or RT-PCR (hTERT, MycN, GAPDH). (B) None of the mice formed tumors. (TIF) [file pone.0140781.s003.tif]
