# Supplementary material for: Role of the YAP Oncoprotein in Priming Ras-Driven Rhabdomyosarcoma
Source: PLoS One. 2015 Oct 23;10(10):e0140781. doi: 10.1371/journal.pone.0140781 (PMC4619859; doi:10.1371/journal.pone.0140781)
Supplement: S1 Table — (PDF) [file pone.0140781.s004.pdf]

Supplemental Table 1

Oligos used for qPCR	
**Oligo**	**Sequence (5'-3')**
YAP1 FW	CCT TCT TCA AGC CGC CGG AG
YAP1 REV	CAG TGT CCC AGG AGA AAC AGC
CTGF FW	GCA GAG CCG CCT GTG CAT GG
CTGF REV	GGT ATG TCT TCA TGC TGG
GAPDH FW	ATG GGG AAG GTG AAG GTC G
GAPDH REV	GGG GTC ATT GAT GGC AAC AAT A
Cyr61 FW	ATT GTA GAA AGG AAG CCT TGC TCA T
Cyr61 REV	TCC AAT CGT GGC TGC ATT AG
MyoD FW	GGT CCC TCG CGC CCA AAA GAT
MyoD REV	CAG TTC TCC CGC CTC TCC TAC
Myogenin FW	CAG TGC ACT GGA GTT CAG CG
Myogenin REV	TTC ATC TGG GAA GGC CAC AGA
Mrf4 FW	CCC CTT CAG CTA CAG ACC CAA
Mrf4 REV	CCC CCT GGA ATG ATC GGA AAC
**Oligos used for semi-quantitative RT-PCR**	
**Oligo**	**Sequence (5'-3')**
hTERT FW	TGG CTG TGC CAC CAA GCA TT
hTERT REV	TTT CCA CAC CTG GTT GC
GAPDH FW	GAG AGA CCC TCA CTG CTG
GAPDH REV	GAT GGT ACA TGA CAA GGT GC
MycN FW	ACC CTC ACC CTA CGT GGA G
MycN REV	CCA AAA GAG GGC AAT ATG G
